# Takes Two to Tangle: A Rare Case of Type IV Dual Left Anterior Descending Artery (LAD) and Dual Ostia of the LAD and Left Circumflex (LCx) Artery

**DOI:** 10.7759/cureus.61953

**Published:** 2024-06-08

**Authors:** Riya George, Linle Hou, Parth Patel, John Makaryus

**Affiliations:** 1 Internal Medicine, Northwell Health, New York City, USA; 2 Cardiology, Northwell Health, Manhasset, USA; 3 Cardiology, North Shore University Hospital, Donald and Barbara Zucker School of Medicine at Hofstra/Northwell, Manhasset, USA

**Keywords:** left anterior descending artery origin from rca, coronary artery angiogram, coronary computed tomography angiogram (ccta), dual ostia, dual lad, caa: coronary artery anomaly

## Abstract

The dual left anterior descending (LAD) artery is a rare anatomic variant of the LAD artery that refers to the duplication of the LAD into a short and long LAD. These two vessels, differentiated based on their lengths, ultimately provide blood supply to the areas normally covered by the LAD. In this case report, we describe an unusual case of a type IV dual LAD system with an additional finding of a separate origin for the short LAD and left circumflex (LCx) artery. These two findings have not been reported together in the literature previously. During diagnostic procedures like coronary angiography or when interpreting cardiac imaging, awareness of these anomalies prevents confusion with pathological conditions such as coronary artery disease or stenosis. Additionally, it is crucial for cardiologists and surgeons to identify these aberrant vessels to avoid any wrongful interventions.

## Introduction

Coronary artery anomalies (CAAs) describe a wide range of rare anatomic variants that are still being studied and discovered due to the sheer variations that we are encountering given advancements in cardiovascular imaging. Embryologically, the evolution of an immature coronary plexus from the sinus venosus, endocardium, and proepicardial cells forms the groundwork of coronary vessel formation. Under the guidance of growth factors and transcription pathways, this immature plexus connects with the aorta forming a primitive coronary artery ostia that further cascades into blood flow initiation and arterial remodeling that ultimately leads to mature coronary arteries. This embryologic complexity involving a myriad of molecular processes, cell migration, and angiogenesis gives us an insight into the various points that could deviate from normal development leading to different types of coronary artery anomalies that have been identified [1,2].

CAAs are broadly categorized into anomalies of origin and course, anomalies of intrinsic coronary artery (CA) anatomy, and anomalies of termination, as it relates to the three epicardial arteries, namely, the right coronary artery (RCA), the left anterior descending (LAD) artery, and the left circumflex (LCx) artery [2,3]. Studies that have reviewed angiograms and autopsies have reported the prevalence of these anomalies to range from 1-5.6% [3]. Though CAAs are congenital malformations, most tend to remain quiescent and are often found incidentally later in adulthood.

Among the wide range of CAAs, the dual LAD system can be categorized under anomalies of intrinsic CA anatomy and is a rare phenomenon referring to the duplication of the LAD into a short and long LAD. Type IV dual LAD is differentiated by the short course of the LAD arising from the left main coronary artery, giving off septal and diagonal branches prior to terminating proximally in the anterior interventricular groove. This type is particularly rare and is notably associated with congenital cardiac conditions such as tetralogy of Fallot or transposition of great arteries. Additionally, this case features an anomaly of origin, with the short LAD and LCx having separate origins. The increasingly widespread use of coronary computed tomography angiography (CCTA) makes the identification of these anomalies more accessible for clinicians. Even though many of these anomalies are found incidentally, recognizing them is crucial to prevent misinterpretation on imaging and avoid unnecessary interventions on these aberrant vessels. Optimal patient care includes the recognition of these CAAs as they influence diagnostic, interventional, and surgical strategies.

This case report highlights a rare type IV dual LAD system, accompanied by the unique finding of separate origins for the short LAD and LCx. This combination of anomalies has not been previously reported, underscoring its significance not only in literature but also in clinical practice. Accurately identifying these anomalies not only aids in differentiation from other coronary artery diseases but is also crucial in the planning of interventional procedures to avoid complications as well as guiding surgical strategies to ensure correct vessel targeting and graft placement.

## Case presentation

A 69-year-old woman of South Asian descent with hypertension (HTN), hyperlipidemia (HLD), hypothyroidism, and asthma presented to the clinic with atypical chest pain (CP). Her symptoms began three months prior to presentation and were associated with mild dyspnea on exertion. Chest pain was described as sharp, intermittent, located in the substernal region, lasting for five minutes per episode, non-radiating, non-pleuritic, 5/10 in severity, not associated with any activity, and no relieving factors. Further review of systems was non-contributory. Vital signs showed a blood pressure of 130/80, heart rate of 78, oxygen saturation of 100% on room air, respiratory rate of 10, and temperature of 97.5 Fahrenheit. Physical examination revealed normal S1/S2, no murmurs were appreciated, lungs were clear to auscultation, no evidence of jugular venous distention, and no lower extremity edema.

**Figure 1:**
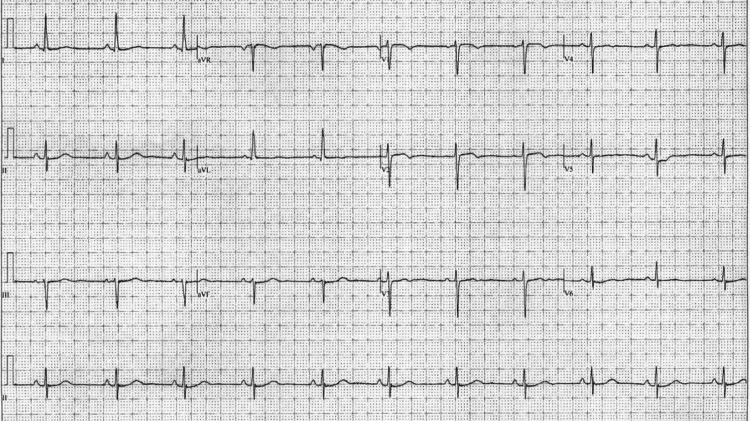
EKG showing normal sinus rhythm with T-wave inversions in leads V1-V3

Electrocardiogram (EKG) showed normal sinus rhythm at 70 beats per minute, normal axis, and new T-wave inversions in leads V1-V3 as shown in Figure 1.

**Figure 2:**
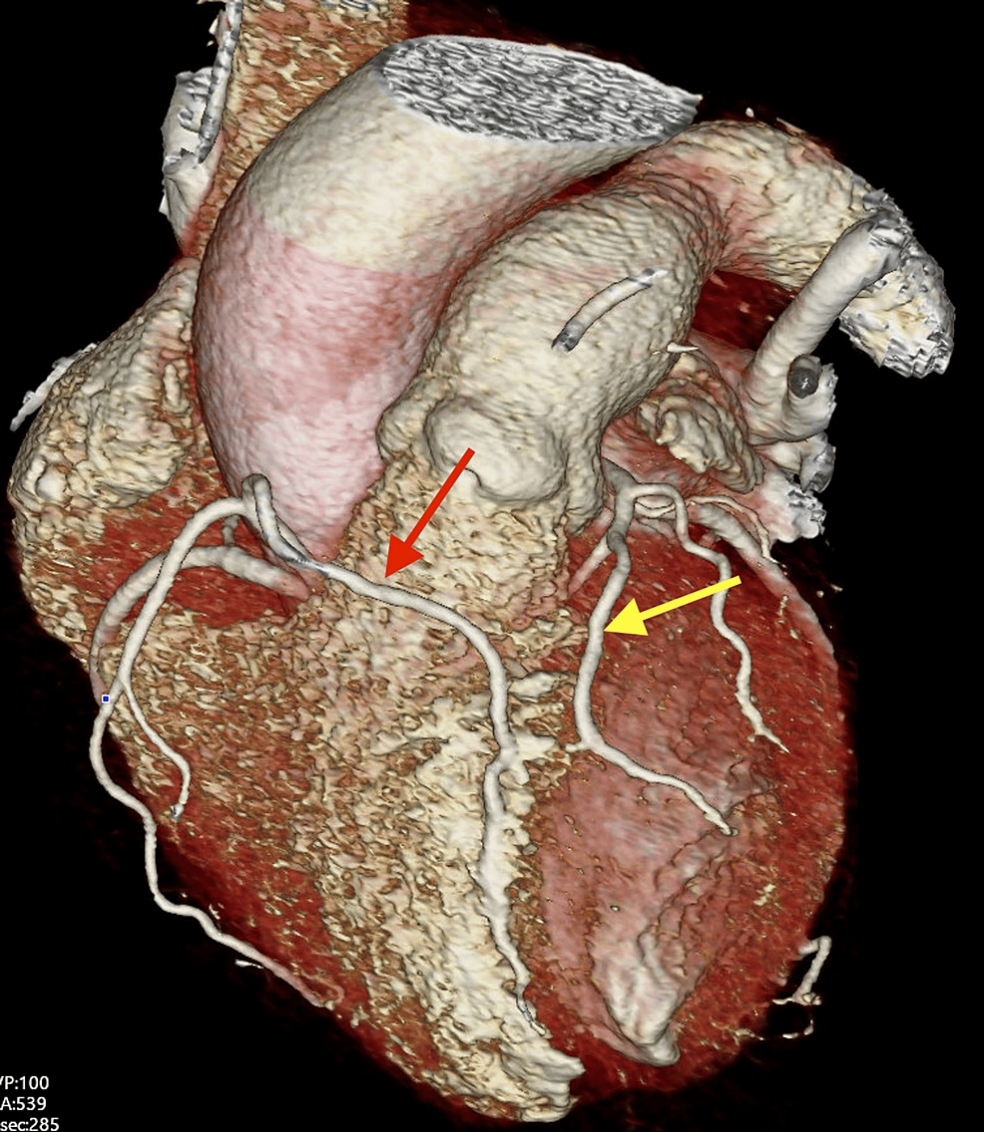
Three-dimensional volume-rendered cardiac computed tomography (CT) depicting long LAD (red arrow) arising from the right sinus of Valsalva and eventually traveling into the anterior interventricular groove. Additionally, the short LAD (yellow arrow) is shown to arise from the left sinus of Valsalva and course along the interventricular septum. LAD: left anterior descending

Given her atypical CP along with new T-wave inversions, there was concern for underlying coronary artery disease (CAD). For risk stratification, she underwent a coronary computed tomography angiography (CCTA), which showed evidence of a dual LAD system with the short LAD arising from the left sinus of Valsalva and terminating on the left ventricular side of the interventricular septum without evidence of obstructive disease. The long LAD was seen to arise from a common ostium with the right coronary artery (RCA) from the right sinus of Valsalva and traveling anteriorly to the right ventricle before entering the anterior interventricular groove. There was no evidence of obstructive disease seen in this long LAD as well, which continued to travel toward the left ventricular apex as shown in Figure 2.

**Figure 3:**
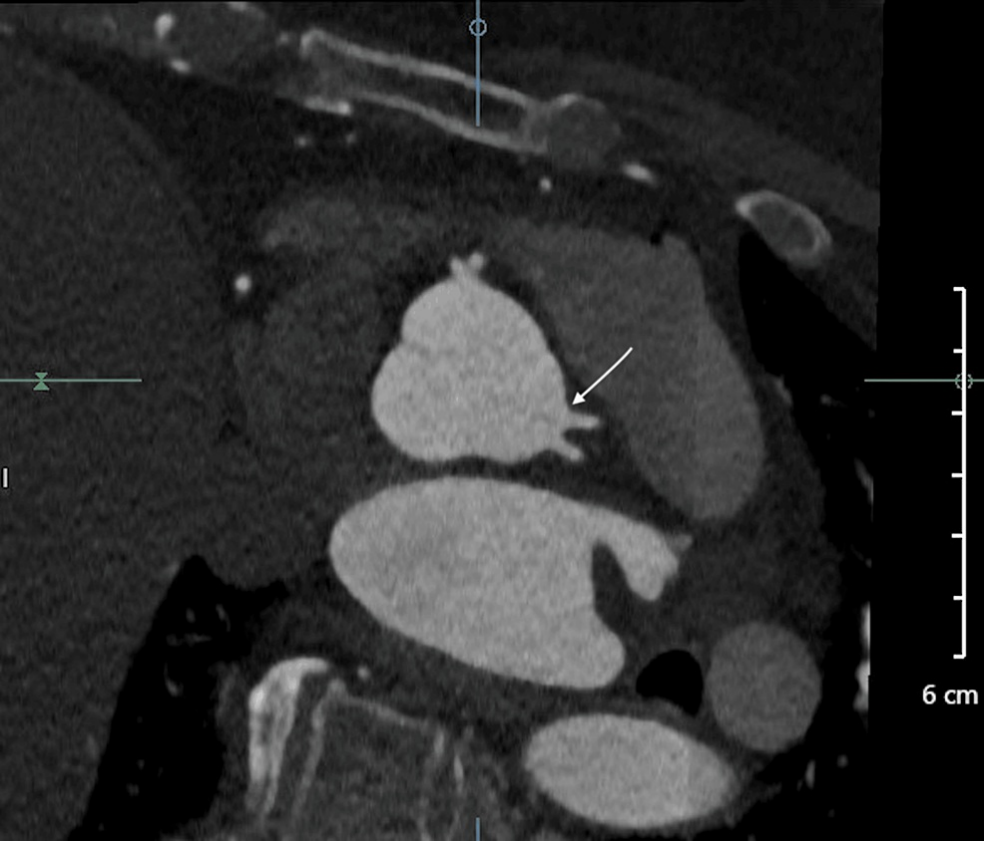
CTA with a white arrow showing the separate ostia arising from the left sinus of Valsalva that is giving rise to a short LAD and LCx CTA: computed tomography angiography; LAD: left anterior descending; LCx: left circumflex

Additionally, the short LAD and LCx were noted to arise from separate but adjacent ostia in the left sinus of Valsalva as shown in Figure 3.

As these vessels did not have any evidence of obstruction or compression from surrounding structures, these anatomic variants could not fully explain this patient's symptoms. Interestingly, CCTA also showed evidence of tracheomalacia, which was followed by pulmonary function testing showing an obstructive pattern of lung disease. To further evaluate this finding as a possible explanation of her dyspnea, a bronchoscopy was done, which showed evidence of significant flattening of the distal tracheal rings and approximately 80% to 90% of airway occlusion throughout the respiratory cycle. She was referred to cardiothoracic surgery, and given that her symptoms were not severe, intervention was deferred. A sleep study was also done, which confirmed obstructive sleep apnea and consequently, she was started on nocturnal continuous positive airway pressure. Ultimately, the patient underwent further medical management with a focus on controlling her risk factors to prevent further complications from her underlying chronic medical conditions. During subsequent outpatient visits, she reported that her chest pain had resolved and her dyspnea had improved.

## Discussion

Normal coronary anatomy and the unique aspect of this case 

In normal coronary anatomy, the left sinus of Valsalva gives rise to the left main stem, which then bifurcates into the LAD and the LCx. The LAD then travels through the anterior interventricular groove, giving off diagonal and septal branches providing blood supply to the territory consisting of the anterolateral myocardium and apex along with the anterior two-thirds of the interventricular septum [4,5].

This case report explores the presence of two separate CAAs that made this case unique. In addition to the separation of the LAD referred to as the dual LAD, we see that there is no common left main coronary; the short LAD and LCx have separate but adjacent origins from the left sinus of Valsalva. Though there are many variants of the dual ostia, for this paper, we will be referring to the separate origins of the LAD and LCx as the dual ostia [6].

Initial identification and epidemiology

From a historical perspective, dual LAD was distinctly first defined in 1983 by Spindola-Franco et al. who described four different types based on the derivation and the course of the short and long LAD [4]. After the publication of this paper, thirteen variants have since been described and proposed in the literature [4,7]. As mentioned previously, type IV dual LAD is differentiated by the short course of the LAD arising from the left main coronary artery, giving off septal and diagonal branches prior to terminating proximally in the anterior interventricular groove. In our patient's case, the short LAD was seen to arise from the left sinus of Valsalva separate from the origin of the LCx due to the absence of a common left main coronary artery. Additionally, the long LAD arises from the right sinus of Valsalva and terminates in the distal anterior interventricular groove [4].

In terms of epidemiology, the prevalence of the dual LAD system was estimated to be 1% with the type IV dual LAD system being recognized as the rarest of the subtypes reportedly found in 0.004% of patients undergoing cardiac catheterization [4,8]. Conversely, a dual ostia is considered one of the most common anomalies with an incidence of 0.41% [9].

Clinical findings

From a clinical perspective, CAAs have been associated with a wide array of presentations, including acute coronary syndrome (ACS), arrhythmias, dyspnea on exertion, or sudden cardiac death in young individuals; interestingly, many CAAs are often found incidentally with no associated symptoms [3]. Dual LAD is usually a hemodynamically insignificant finding that is only prominent in variants that have structures in close proximity or tortuous pathways prone to compression. Specifically, the type IV dual LAD has largely been reported to be an incidental finding but there are rare reports associating it with stable angina, ACS, and heart failure [9,10]. In a case report by Papadopoulos et al., a patient with subacute symptoms of angina pectoris and a positive stress test was found to have a type IV dual LAD system. His symptoms were thought to be due to systolic compression of an intraseptal segment. This article highlights the importance of the identification of the dual LAD system and how it relates to the surrounding structures, which could offer an explanation of atypical symptomatology. It also highlights how easy it is to misinterpret this variation of the LAD as having total occlusion, thereby potentially adversely affecting management [11]. Additionally, the dual ostia is considered a benign anatomic anomaly with no symptoms described in literature thus far.

Diagnosis

Though the clinical significance of these anomalies requires further exploration, the technological advances made in the field of cardiac imaging have helped in the identification of these anomalies at an expedited rate. Invasive coronary angiography (ICA) was previously considered to be the most definitive tool in identifying CAAs, however, CCTA is now considered the gold standard for the diagnosis of CAAs [3]. CCTA allows for non-invasive identification in addition to mapping an accurate course for these vessels while providing information regarding the surrounding structures.

Clinical relevance

In our case, the patient’s symptoms are not explained by this anatomic variant given that her coronaries showed a non-obstructive pattern and there was no compression of the coronaries by the surrounding structures. Though the clinical significance of these anomalies is not fully described, accurate identification can prevent interventions in the wrong vessels. Incorrect placement during an arteriotomy due to improper identification or even grafting of a short LAD due to its misinterpretation as a total occlusion can be detrimental to patient care [12]. Cardiologists and surgeons should therefore be well-versed in the identification of these anomalies.

## Conclusions

This case explores the concomitant presence of a rare anatomical abnormality with a relatively common anomaly. A type IV dual LAD along with a dual ostia has never been reported together in the literature previously. CCTA is considered the gold standard for the identification and delineation of these abnormalities. The continued advancements in imaging will bring to the forefront many more variations and combinations of CAAs, highlighting the need for clinicians to be familiar with these anomalies.

It remains pertinent for clinicians to accurately identify variations of coronary vessels, as it can guide interventions in symptomatic patients and ensure appropriate management. Beyond individualized patient care, developing databases that can identify these anomalies and chart clinical events, interventions, and outcomes can help future clinicians answer challenging questions posed by these anomalies. This can ultimately improve diagnostic accuracy, which will inevitably translate into personalized treatment plans and better patient outcomes.
